# Leucine-rich alpha-2-glycoprotein 1 and angiotensinogen as diagnostic biomarkers for Kawasaki disease

**DOI:** 10.1371/journal.pone.0257138

**Published:** 2021-09-09

**Authors:** Masakatsu Yanagimachi, Sayaka Fukuda, Fumiko Tanaka, Mari Iwamoto, Chiho Takao, Kunihiro Oba, Natsuko Suzuki, Koji Kiyohara, Dai Kuranobu, Norimasa Tada, Ayako Nagashima, Taku Ishii, Yoko Ino, Yayoi Kimura, Nobutoshi Nawa, Takeo Fujiwara, Takuya Naruto, Tomohiro Morio, Shouzaburo Doi, Masaaki Mori

**Affiliations:** 1 Department of Pediatrics, Tokyo Medical and Dental University, Tokyo, Japan; 2 Department of Hematology/Oncology, Kanagawa Children’s Medical Center, Yokohama, Japan; 3 Department of Pediatrics, Saiseikai Yokohama-shi Tobu Hospital, Yokohama, Japan; 4 Department of Pediatrics, Saiseikai Yokohama-shi Nanbu Hospital, Yokohama, Japan; 5 Department of Pediatrics, Showa General Hospital, Tokyo, Japan; 6 Department of Pediatrics, Japanese Red Cross Musashino Hospital, Tokyo, Japan; 7 Department of Pediatrics, Tokyo-Kita Medical Center, Tokyo, Japan; 8 Department of Pediatrics, Kawaguchi Municipal Medical Center, Saitama, Japan; 9 Department of Pediatrics, Tsuchiura Kyodo General Hospital, Ibaraki, Japan; 10 Advanced Medical Research Center, Yokohama City University, Yokohama, Japan; 11 Department of Global Health Promotion, Tokyo Medical and Dental University, Tokyo, Japan; 12 Department of Lifetime Clinical Immunology, Graduate School of Medical and Dental Sciences, Tokyo Medical and Dental University, Tokyo, Japan; 13 Department of Community Pediatrics, Perinatal, and Maternal Medicine, Tokyo Medical and Dental University, Tokyo, Japan; H Lee Moffitt Cancer Center and Research Institute, UNITED STATES

## Abstract

**Objective:**

Kawasaki disease (KD) is a systemic vasculitis in childhood that can lead to coronary artery lesions (CALs). Although early diagnosis and treatment is important for preventing KD patients from development of CALs, diagnosis depends on the clinical features of KD. We studied the usefulness of leucine-rich alpha-2-glycoprotein 1 (LRG1) and angiotensinogen (AGT), previously reported as KD-related proteins, for KD diagnosis and estimation of intravenous immunoglobulin (IVIG) efficacy.

**Methods:**

We undertook a prospective cohort study with patients having two or more KD symptoms in multiple centers in Japan, between July 2017 and February 2019.

**Results:**

Two hundred forty-two patients were included. In multivariable analysis, one unit increase in LRG1 was associated with higher odds of KD diagnosis (Odds ratio [OR] 1.02 [95% confidence interval (CI) 1.001–1.03]). Double-positivity for AGT (≥ 26 μg/mL) and LRG1 (≥ 123.5 μg/mL) was an independent biomarker for KD diagnosis in both the total cohort and the subgroup of patients with two to four KD symptoms (OR 5.01 [95% CI 1.86–13.50] and 3.71 [95% CI 1.23–11.16], respectively). There was no association between LRG1/AGT and IVIG efficacy.

**Conclusion:**

Double-positivity for LRG1 and AGT is an biomarker for KD diagnosis, especially useful in diagnosing incomplete KD from non-KD. Future studies with larger cohorts should seek to determine whether LRG1 and AGT are valuable as definitive data referred at the diagnosis of KD and for estimating the risk of CALs.

## Introduction

Kawasaki disease (KD) is a systemic vasculitis in childhood that can lead to coronary artery lesions (CALs) [1–3]. Since the introduction of intravenous immunoglobulin (IVIG) therapy in the latter half of the 1980s, the onset rate of CALs in KD patients has decreased to 9.3% at the acute phase and 2.8% at the recovery phase [4]. Although early diagnosis and treatment is important for preventing KD patients from developing CALs, diagnosis of KD depends on the disease’s clinical features and the provider’s experience. Therefore, it is sometimes difficult to make a definitive diagnosis. Diagnosis of complete KD is based on five or more KD symptoms, including persistent fever, acute non-purulent cervical lymphadenopathy, hyperemia of the bulbar conjunctiva, strawberry tongue, redness and cracking of the lips, hard edema of the hands and feet, and angiitis symptoms such as redness of finger tips [2]. Patients without five features but with supportive lab studies and no other diagnosis were considered for incomplete KD [5]. Importantly, incomplete KD leads to development of CALs as frequently as complete KD with five or more KD symptoms [6, 7]. Supplemental laboratory data such as leukocytosis with neutrophilia and immature forms, hypoalbuminemia, and hyponatremia are sometimes referred as “significant findings” in Japanese and the American Heart Association (AHA) KD guidelines [5, 8]. We previously identified four KD-related proteins, leucine-rich alpha-2-glycoprotein 1 (LRG1), angiotensinogen (AGT), lipopolysaccharide-binding protein (LBP), and retinol-binding protein 4 (RBP4) [9]. In particular, LRG1 is associated with fibrosis, blood vessel remodeling, and neutrophil differentiation, and has been reported as a biomarker for CALs associated with KD [10–12]. AGT is a key enzyme of the renin–angiotensin system that is associated with vascular injury, such as atherosclerosis and thrombosis [13]. We performed a prospective cohort study to evaluate the usefulness of LRG1 and AGT for KD diagnosis and estimation for IVIG efficacy.

## Patients and methods

We conducted a prospective cohort study at eight tertiary pediatric hospitals in Japan. Patients who had two or more KD symptoms between July 1, 2017 and February 28, 2019 were included. KD was diagnosed according to the Japanese diagnostic guideline for KD [8]. Complete KD was defined as the presence of five or six KD symptoms, and incomplete KD was defined as the presence of four or fewer KD symptoms, excluding KD-mimic diseases such as streptococcal infections, adenovirus infections, and Stevens–Johnson syndrome. Supplemental laboratory data used for KD diagnosis include neutrophilia, low hematocrit, low platelet count, elevated serum transaminases (e.g., alanine aminotransferase, ALT), hypoalbuminemia, hyponatremia, and elevated CRP.

The study was approved by the ethics committee or institutional review board at each institution (the Institutional Review Boards at Tokyo Medical and Dental University, M2017-017). All patients or their guardians provided written informed consent. This trial is registered with the University Hospital Medical Information Network clinical trials registry (UMIN000028340).

### Inclusion criteria

Patients who were suspected of having KD by provider with two or more symptoms of clinical criteria for KD were included to this study.

### Exclusion criteria

Patients meeting the following criteria were excluded: 1) diagnosis of collagen disease such as juvenile idiopathic arthritis, 2) diagnosis of autoinflammatory syndrome, such as Cryopyrin-associated periodic syndrome, and 3) receiving systemic administration of immunosuppressant or steroid, except for inhaled steroid therapy or anti-allergy medicine. 4) informed consents were not acquired.

### Study design

**Fig 1:**
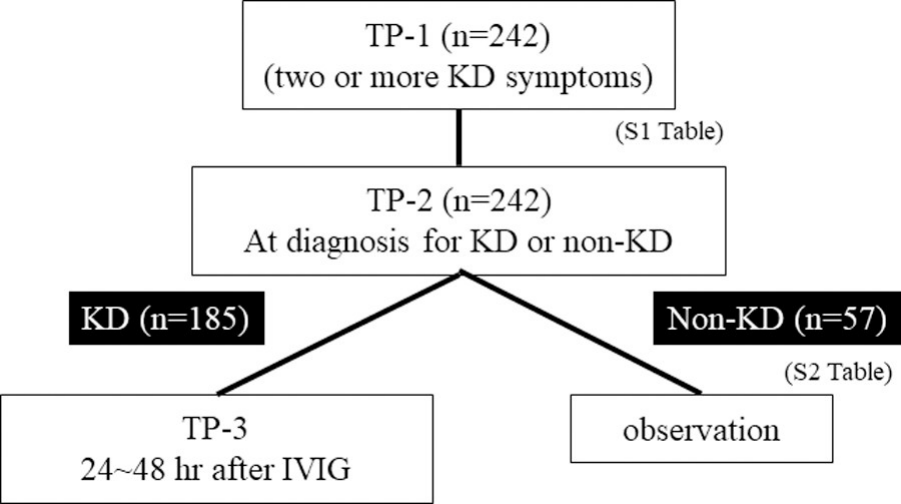
Flow chart of the study. TP-1: when a patient with two or more KD symptoms was suspected as having KD; TP-2: when a patient was diagnosed as KD or non-KD by a provider; and TP-3: 24–48 hours after first IVIG in a patient with KD.

Participants were evaluated by clinical examination and laboratory test (including LRG1 and AGT) at the following time points: TP-1, when a patient with two or more KD symptoms was suspected of having KD by a provider; TP-2, when a patient was diagnosed as having KD or non-KD by a provider; and TP-3, 24–48 hours after administration of the first dose of IVIG (2 g/kg patient’s body weight) (Fig 1). The day of collecting samples and data were dependent on a provider’s clinical decision.

The efficacy of IVIG was defined as reduction of the patient’s temperature below 37.5°C 24–48 hours after the first IVIG treatment without exacerbation of KD symptoms or additional IVIG treatment. The following patients were included in ineffective to IVIG: patients who were treated with second IVIG treatment by a provider judgment because of residual KD symptoms except for fever or recurrent fever 2-4days after the first IVIG treatment completed.

### ELISA for LRG1 and AGT

The following ELISA kits were used: LRG1 ELISA kit (Cosmic Corporation, Tokyo, Japan, product number; T131C100) and Human Total Angiotensinogen (AGT) Assay Kit (Immuno-Biological Laboratories, Gunma, Japan, product number; 27412).

### Statistical analysis

Differences of continuous values between two groups were compared by Mann–Whitney test, and differences among three groups were compared by Kruskal–Wallis test. Differences of nominal variables among two or three groups were compared by chi-square test. Odds ratios (ORs) and 95% confidence intervals (CIs) were calculated by univariable and multivariable logistic regression models. Coefficient of correlation was calculated by Pearson correlation coefficient analysis. P-values <0.05 were considered statistically significant. The sample size was calculated based on the assumed prevalence of Kawasaki disease (50% to 65%) in a tertiary hospital patient population of those with double-negative results for the two markers, assuming an odds ratio of 3.5 to 8.0 for both positive results compared with both negative results for the two markers. Using a two-tailed test with a significance level of 0.05 and a sample size of 125, a statistical power of 80% was obtained. Multiplied by 1.5 to account for the number of patients with a single positive result, and with a margin of 10%, a sample size of 209 or more was estimated to be sufficient.

All analyses were performed with STATA SE statistical package, version 14 (StataCorp LP, College Station, TX, USA) and EZR (Saitama Medical Center, Jichi Medical University, Saitama, Japan), a graphical user interface for R (The R Foundation for Statistical Computing, Vienna, Austria) [14].

## Result

Two hundred forty-two patients with more than two KD symptoms were included. The clinical data and ELISA measurements for each patient were indicated in S1 Table. Clinical and laboratory data at TP-1 (when a provider suspected that the patient had KD) were summarized in Table 1. One hundred eighty-five KD patients (123 complete KD and 62 incomplete KD) and 57 non-KD patients were included in the study. Median day of TP-1 was later in patients with incomplete KD than in patients with complete KD or non-KD (p = 0.02) (S1 Fig). Patients who had two to five KD symptoms at TP-1 were finally diagnosed as incomplete KD, complete KD, or non-KD based on a provider’s clinical judgment at TP-2. In 106 patients, the diagnosis was made at TP1, therefore TP-2 was the same timing of TP-1 in those patients. A total of 179 KD patients (121 complete KD and 58 incomplete KD) were treated with IVIG or aspirin. Thirteen patients were concurrently administered steroid with IVIG based on a provider’s judgment. Six KD patients were treated without IVIG (e.g., aspirin only). Therefore, 166 KD patients were received only IVIG and aspirin as the initial treatment. Fifty-seven patients were diagnosed as non-KD and treated only with antimicrobial drug or supportive care such as antipyretic medication. Non-KD patients included those with pyogenetic cervical lymphadenitis or viral infections (S2 Table).

**Table 1:** Patient characteristics.

	Diagnosis	Non-KD	Incomplete KD	Complete KD	
	(n)	(57)	(62)	(123)	P-value
Age		2.0 [0.0, 9.0]	2.0 [0.0, 13.0]	1.0 [0.0, 13.0]	0.28
Sex n (%)	male	34 (59.7)	34 (54.8)	72 (58.5)	0.85
	female	23 (40.4)	28 (45.2)	51 (41.5)	
Day at TP1		4.0 [1.0, 14.0]	5.0 [2.0, 12.0]	4.0 [2.0, 9.0]	0.02
Number of KD-symptoms	2	17 (29.8)	9 (14.5)	7 (5.7)	<0.01
at TP1 n (%)	3	17 (29.8)	19 (30.7)	2 (1.6)	
	4	18 (31.6)	31 (50.0)	17 (13.8)	
	5	5 (8.8)	3 (4.8)	57 (46.3)	
	6	0 (0.0)	0 (0.0)	40 (32.5)	
Past history of KD n (%)	no	53 (93.0)	61 (98.4)	119 (96.8)	0.28
	yes	4 (7.0)	1 (1.6)	4 (3.3)	
Family history of KD n (%)	no	55 (96.5)	60 (96.8)	114 (94.3)	0.68
	yes	2 (3.5)	2 (3.2)	7 (5.7)	
KD, Kawasaki disease					

TP1, Time point 1 when a patient with two or more KD-symptoms was suspected as having KD.

### Usefulness of AGT and LRG1 for KD diagnosis

To evaluate the usefulness of supplemental laboratory data and serum levels of two KD-related proteins in KD diagnosis, we performed univariable analysis. All factors analyzed were statistically different between KD (combined complete and incomplete KD) and non-KD patients at TP-1 (S3 Table and S2 Fig).

LRG1 was associated with CRP in the KD (combined complete and incomplete KD) (r^2^ = 0.46 [95% CI 0.32–0.57] p<0.01) and non-KD (r^2^ = 0.70 [95% CI 0.53–0.81] p<0.01) subgroups. AGT was not associated with CRP in the KD (r^2^ = 0.09 [95% CI -0.05–0.23] p = 0.21) and non-KD (r^2^ = 0.16 [95% CI -0.1–0.41] p = 0.22) subgroups. We added figures showing the association of CRP with AGT and LRG1 in the KD and non-KD subgroups (S3 Fig)

In multivariable analysis, high percentage of neutrophils in white blood cells, low degrees of hematocrit, albumin and CRP, and high levels of LRG1 and were associated with KD diagnosis in this cohort. LRG1 was associated with having KD (OR 1.02, 95% CI 1.001–1.03, p = 0.04) and ATG was not (OR 1.04, 95% CI 0.99–1.09, p = 0.08) (Table 2).

**Table 2:** Multivariable analysis of laboratory data for diagnosis of KD.

	Odds ratio	95% CI	P-value
Neutrophil (%)	1.04	1.00–1.13	<0.01
Hematocrit (%)	0.82	0.70–0.97	0.02
Platelet count (10*4/μL)	1.01	0.98–1.10	0.49
Albumin (g/dL)	0.11	0.03–0.36	<0.01
ALT (IU/L)	1.00	0.99–1.02	0.59
Na (mEq/L)	0.87	0.74–1.01	0.12
CRP (mg/dL)	0.89	0.78–0.99	0.048
AGT (μg/mL)	1.04	0.99–1.10	0.08
LRG1 (μg/mL)	1.02	1.00–1.03	0.04

KD, Kawasaki disease (combined complete and incomplete Kawasaki disease). CI, confidence interval.

For KD diagnosis, cut-off values of serum AGT (26.0 μg/mL) and LRG1 (123.5 μg/mL) levels were determined by the Stata roctab command to output a table of sensitivity and specificity for each threshold, and chose the threshold that maximized both. In KD diagnosis, the sensitivity and specificity of one or more positive cut-off values of AGT and LRG1 were 0.83 and 0.39, respectively. Positive and negative predictive values were 0.81 and 0.41, respectively. According to AHA guideline, CRP above 3 mg/dl is one of the supplemental data used for diagnosis of incomplete KD [5]. Next, we evaluated the utility of CRP and double positivity for LRG1 and AGT as the supplemental data in the diagnosis of incomplete KD in our cohort. In our cohort, CRP (≥3.0 mg/dL) and double-positivity for AGT (≥26 μg/mL) and LRG1 (≥123.5 μg/mL) were independent biomarkers for KD diagnosis (Table 3A). These diagnostic values were kept in the subgroup analysis in patients with two to four KD symptoms (Table 3B). Double-positivity for AGT and LRG1 was an independent biomarker for KD diagnosis in the overall cohort (n = 242) (OR 5.01 [95% CI 1.86–13.50]), and in the subgroup having two to four KD symptoms (n = 137) (OR 3.71 [95% CI 1.23–11.16]). Odds ratio for the double-positivity for AGT and LRG1 was higher than that of CRP in diagnosing KD from non-KD.

**Table 3:** Diagnostic values of AGT, LRG1, and CRP for KD.

A) All patients (n = 242)					
		Univariable model		Multivariable model	
		Odds ratio	95% CI	Odds ratio	95% CI
AGT+LRG	Ref (double negative)				
	Single positive	1.87	0.93–3.74	1.35	0.64–2.84
	Double positive	7.56	2.94–19.46	5.01	1.86–13.50
CRP		4.17	2.18–7.97	3.07	1.53–6.17
B) Patients with 2–4 KD-symptoms (n = 137)					
		Univariable model		Multivariable model	
		Odds ratio	95% CI	Odds ratio	95% CI
AGT+LRG	Ref (double negative)				
	Single positive	1.18	0.51–2.73	0.95	0.39–2.30
	Double positive	5.0	1.74–14.34	3.71	1.23–11.16
CRP		2.92	1.34–6.35	2.34	1.02–5.38

KD, Kawasaki disease, Cut-off values of serum AGT, LRG1 and CRP; 26.0 μg/mL, 123.5 μg/mL and 3.0 mg/dL, respectively, CI, confidence interval.

### Usefulness of AGT and LRG1 for estimating IVIG efficacy

Among 185 KD patients, 166 (108 complete KD and 58 incomplete KD) were treated with IVIG and aspirin without other drugs such as steroids. In these 166 patients treated by IVIG concomitantly with aspirin, 113 (68.1%) were afebrile 24–48 hours after IVIG without exacerbation of KD symptoms or additional treatment other than aspirin. Fifty-three patients (31.9%) were treated with the second IVIG treatment because of having fevers above 37.5°C and/or residual KD symptoms 24–48 hours after the initial IVIG treatment or having recurrent fever 2–4 days after the first IVIG treatment completed. There was no difference in IVIG efficacy between complete and incomplete KD patients. High percentages of neutrophils in white blood cells, high ALT and CRP levels, hyponatremia, and days after fever onset were associated with ineffectiveness of IVIG in univariable analysis (S4 Table). LRG1 was also associated with ineffectiveness of IVIG in univariable analysis (p = 0.04), but AGT was not (p = 0.29) in univariable analysis.

In multivariable analysis, high percentage of neutrophils in white blood cells was associated with ineffectiveness of IVIG, but AGT and LRG1 were not (Table 4).

**Table 4:** Multivariable analysis of clinical and laboratory data in relation to IVIG effectiveness.

	Odds ratio	95% CI	P-value
Age	1.14	0.93–1.41	0.22
Day at IVIG after fever onset	1.05	0.76–1.44	0.76
Neutrophil (%)	0.95	0.91–0.99	<0.01
Hematocrit (%)	0.95	0.82–1.11	0.55
Platelet count (10*4/μL)	0.99	0.96–1.03	0.61
Albumin (g/dL)	1.11	0.34–3.56	0.87
ALT (IU/L)	1.00	0.99–1.00	0.07
Na (mEq/L)	1.10	0.93–1.30	0.27
CRP (mg/dL)	0.94	0.85–1.05	0.26
AGT (μg/mL)	0.98	0.95–1.02	0.32
LRG1 (μg/mL)	1.00	0.99–1.02	0.27

CI, confidence intervals.

## Discussion

We validated that LRG1 was a biomarker for differential diagnosis of KD from KD-mimic diseases in this study (Table 2). In addition, double-positivity for LRG1 and AGT was a useful biomarker for KD diagnosis in the clinically challenging setting of differentiating between incomplete KD and non-KD in patients with two to four KD symptoms (Table 3).

In previous work, we identified KD-related proteins, including LRG1, AGT, LBP, and RBP4, by proteomic analysis of the serum of KD patients before and after IVIG treatment [9]. Among the KD-related proteins, LRG1 and AGT are associated with vasculitis and CALs in KD patients [11, 15]. In this study, we analyzed whether LRG1 and AGT were useful biomarkers for diagnosis of KD and estimation of IVIG efficacy.

Since its discovery about 60 years ago, KD has been diagnosed based on its clinical manifestations [3]. Although the etiology of KD has remained unexplained, activation of the immune system, mainly of innate immunity, and an increase in production of inflammatory cytokines such as tumor necrosis factor are considered central to the pathological condition [16]. CAL is the most important complication of KD. Early precise diagnosis and unhesitating IVIG treatment is the key to preventing KD patients from developing CALs [6]. However, the diagnosis of KD still depends on clinical signs and symptoms that are empirical, subjective, and nonspecific. Therefore, an objective and specific biomarker for KD diagnosis is required. Candidate diagnostic biomarkers for KD, such as prostaglandin E2, transthyretin, and N-terminal probrain natriuretic peptide (NT-proBNP), have been reported, but there is no gold-standard test used as a reference for diagnosis of KD [17].

We found double-positivity for LRG1 and AGT was a useful biomarker for KD diagnosis in the clinically challenging setting of differentiating between incomplete KD and non-KD in patients with two to four KD symptoms (Table 3). In this study, we determined the optimal cutoff value. However, this cutoff value will need to be validated in future studies. A diagnostic algorithm for incomplete KD using CRP (≥3 mg/dl), proposed by the AHA guideline, has yielded contentious and divisive results from validation studies [18, 19]. A KD diagnostic biomarker that is independent of CRP is necessary because CRP is sometimes high in patients with KD-mimic diseases, such as adenovirus infection.

LRG1 and AGT have potential as biomarkers for KD diagnosis (Tables 2 and 3). LRG1 was recently reported as a biomarker for the activity of inflammatory bowel disease [20]. AGT is an important factor in the renin–angiotensin system that is associated with vascular injury [13]. Notably in this regard, AGT is negatively regulated by NT-proBNP, a candidate diagnostic biomarker for KD [21]. In our cohort that had inadequate data, we observed no association between AGT and NT-proBNP (r^2^ = 0.12 [95% CI 0.08–0.31] p = 0.26) (S4 Fig). Future studies should investigate the association between AGT and NT-proBNP in KD patients, especially those with CALs.

This study has some limitations. First, we could not determine the usefulness of LRG1 and AGT for estimating the risk of CALs, because this study include only seven patients developed aneurysm. Future studies with a larger cohort should investigate whether LRG1 and AGT have value for determining the risk of CALs. Second, this study did not define the day of collecting samples or data; rather they were dependent on a provider’s clinical decision. However, there were no correlations between the day and LRG1/AGT/CRP in the KD and non-KD subgroups. Third, the definition of efficacy of IVIG was different from AHA guideline, because the study design permitted that a provider decided the additional treatment for non-responder anytime 24 hours after the initial IVIG treatment. Patients, who were treated with second IVIG treatment because of residual KD symptoms except for fever or recurrent fever 2-4days after the first IVIG treatment completed, were also included as ineffective for IVIG. Therefore, a future study with a larger cohort of rigorous definition of IVIG efficacy is needed. The hospitals included in the study were tertiary pediatric hospitals that take in patients with suspected KD from primary clinics and secondary hospitals. Therefore, the rate of negative diagnosis for KD was relatively low. We hope to clarify whether double positivity for LRG1 and AGT is useful for KD diagnosis in other clinical settings, such as primary clinics.

## Conclusion

Double-positivity for LRG1 and AGT is useful for KD diagnosis. Future studies with a larger cohort should investigate whether LRG1 and AGT have value as supplemental laboratory data for determining the risk of CALs and whether early diagnosis of KD using double-positivity for LRG1 and AGT can prevent KD patients from developing CALs.

## Supporting information

S1 Fig Comparison of the day of TP-1 among clinical groups. Comparison of the day of TP-1 in complete-KD, incomplete-KD, and non-KD patients (p = 0.02).
(TIF)

S2 Fig Comparison of each parameters in S3 Table between the KD and non-KD subgroups. (TIF)

S3 Fig Correlation between CRP and LRG1/AGT. Correlation between CRP and LRG1 in (A) KD and (B) Non-KD patients at TP1. Correlation between CRP and AGT in (C) KD and (D) Non-KD patients at TP1.
(TIF)

S4 Fig Correlation between AGT and NT-proBNP. (TIF)

S1 Table Clinical data and ELISA measurements for each patient. (XLSX)

S2 Table Differential diagnosis of non-KD patients in this study. (XLSX)

S3 Table Univariable analysis of laboratory data for diagnosis of KD. (XLSX)

S4 Table Univariable analysis of clinical and laboratory data in relation to IVIG effectiveness. (XLSX)

## Abbreviations

AGT: angiotensinogen
AHA: the American Heart Association
ALT: Alanine aminotransferase
CALs: coronary artery lesions
CI: confidence interval
Ht: hematocrit
IVIG: intravenous immunoglobulin
KD: Kawasaki disease
LBP: lipopolysaccharide-binding protein
LRG1: leucine-rich alpha-2-glycoprotein 1
NT-proBNP: N-terminal probrain natriuretic peptide
OR: Odds ratio
RBP4: retinol-binding protein 4
