# Supplementary figures and images for: Leucine-rich alpha-2-glycoprotein 1 and angiotensinogen as diagnostic biomarkers for Kawasaki disease
Source: PLoS One. 2021 Sep 9;16(9):e0257138. doi: 10.1371/journal.pone.0257138 (PMC8428710; doi:10.1371/journal.pone.0257138)

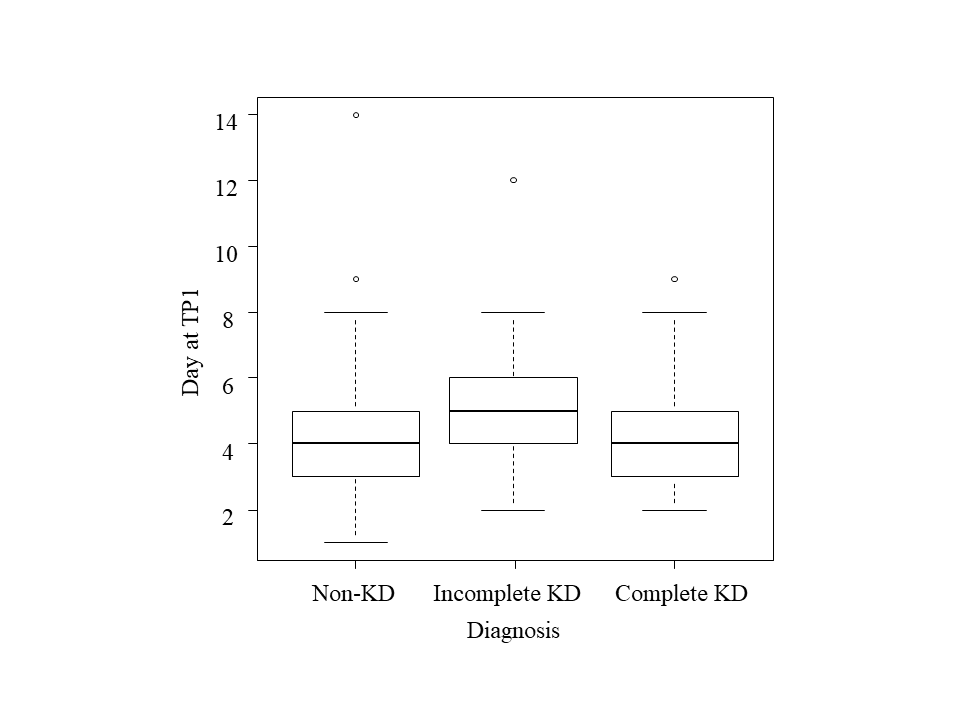

Supplement: S1 Fig — Comparison of the day of TP-1 in complete-KD, incomplete-KD, and non-KD patients (p = 0.02). (TIF) [file pone.0257138.s001.tif]

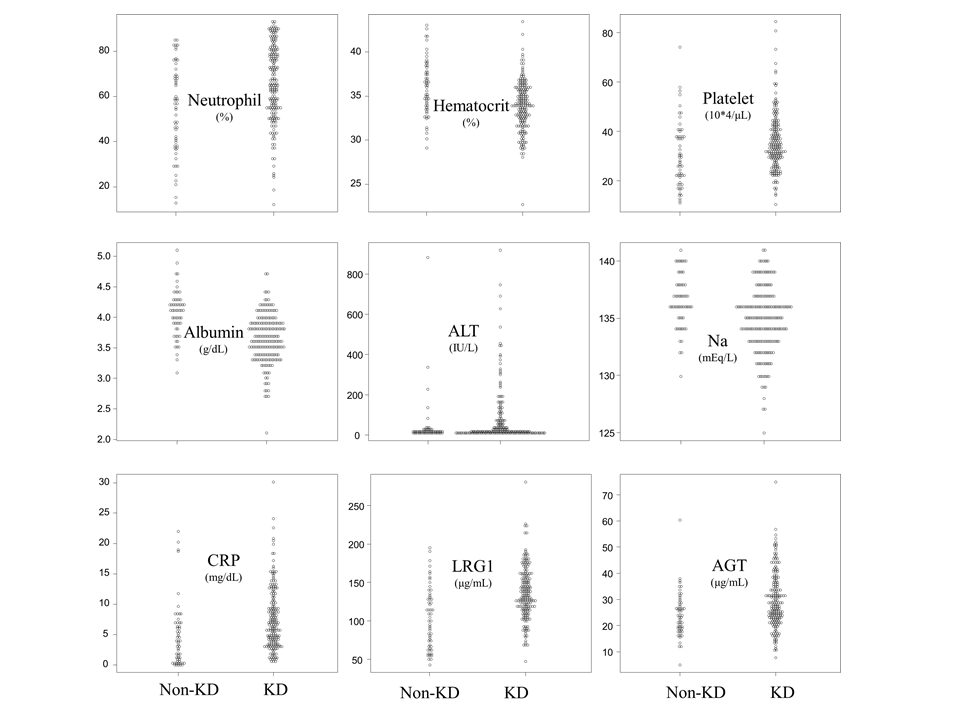

Supplement: S2 Fig — (TIF) [file pone.0257138.s002.tif]

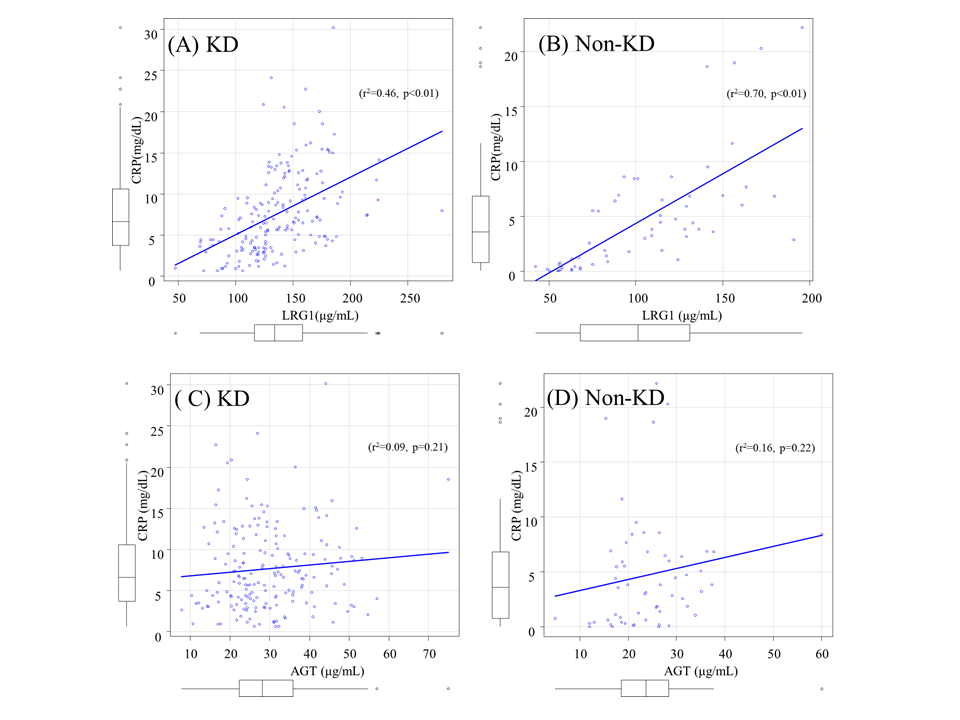

Supplement: S3 Fig — Correlation between CRP and LRG1 in (A) KD and (B) Non-KD patients at TP1. Correlation between CRP and AGT in (C) KD and (D) Non-KD patients at TP1. (TIF) [file pone.0257138.s003.tif]

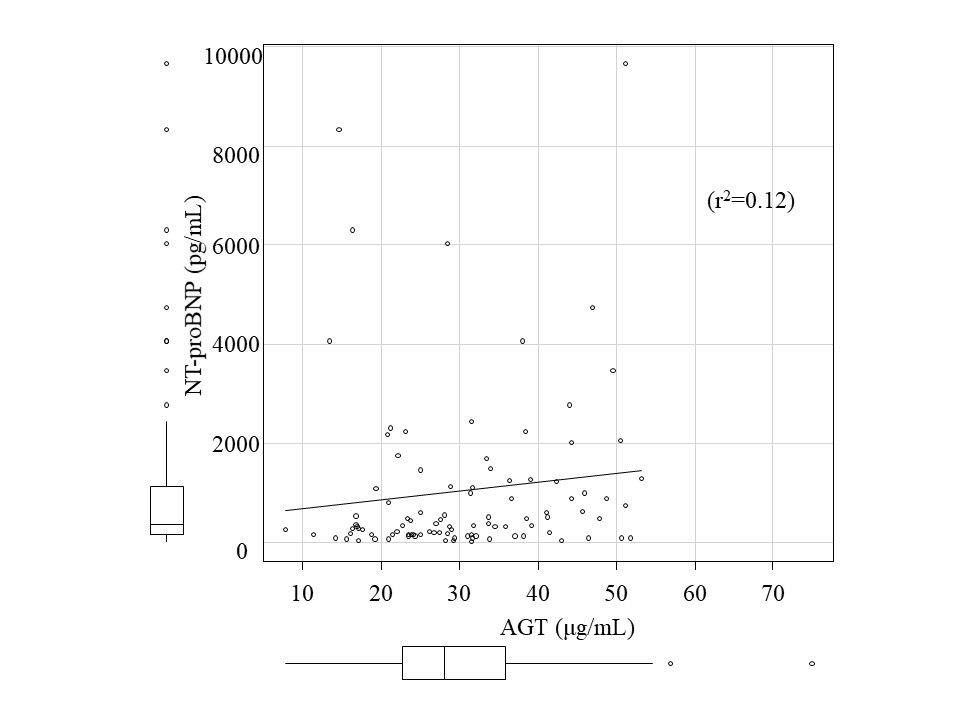

Supplement: S4 Fig — (TIF) [file pone.0257138.s004.tif]
